# Effectiveness of Percutaneous Electrolysis in Supraspinatus Tendinopathy: A Single-Blinded Randomized Controlled Trial

**DOI:** 10.3390/jcm9061837

**Published:** 2020-06-12

**Authors:** Manuel Rodríguez-Huguet, Jorge Góngora-Rodríguez, Pablo Rodríguez-Huguet, Alfonso Javier Ibañez-Vera, Daniel Rodríguez-Almagro, Rocío Martín-Valero, Ángeles Díaz-Fernández, Rafael Lomas-Vega

**Affiliations:** 1 Department of Nursing and Physiotherapy, University of Cádiz, 11009 Cádiz, Spain; manuel.rodriguez@uca.es; 2 Hospital de La Línea de la Concepción, 11300 Cádiz, Spain; 3 Policlínica Santa María Clinic, 11008 Cádiz, Spain; jorgem.gongora@gmail.com; 4 Andalusian Regional Public Health Service Hospital of Jerez de la Frontera, 11407 Cadiz, Spain; prhuguet@hotmail.com; 5 Department of Health Sciences, University of Jaén, Campus las Lagunillas, 23071 Jaen, Spain; dralmagro4@gmail.com (D.R.-A.); andiaz@ujaen.es (Á.D.-F.); rlomas@ujaen.es (R.L.-V.); 6 Department of Physiotherapy, Faculty of Health Sciences, University of Málaga, 29071 Málaga, Spain; rovalemas@uma.es

**Keywords:** supraspinatus tendinopathy, percutaneous electrolysis, trigger point dry needling, shoulder pain

## Abstract

Supraspinatus tendinopathy is one of the most common causes of shoulder pain. Many studies support conservative treatments such as exercise, trigger point dry needling or corticosteroid injections. Otherwise, a minimally invasive approach with percutaneous electrolysis (PE) has also been used successfully in shoulder pain, although evidence about its long-term effects is scarce. The aim of this trial was to determine the effects of PE on supraspinatus tendinopathy compared with trigger point dry needling (TDN). Thirty-six patients with supraspinatus tendinopathy were randomly assigned to either a PE group (*n* = 18) or a TDN group (*n* = 18). Both groups also performed eccentric exercises. The main outcome to be measured was the Numerical Pain Rating Scale (NPRS), but the shoulder range of motion (ROM) and trigger point pressure pain threshold (PPT) were also considered. A one-year follow-up was conducted. Significant differences favoring the PE group were found regarding pain at one-year follow-up (*p* = 0.002). The improvement achieved in the PE group was greater in the NPRS (*p* < 0.001), proximal PPT, middle PPT, distal PPT (all *p* < 0.001) and ranges of movement. PE seems to be more effective than TDN in relieving pain and improving ROM and PPT supraspinatus values in patients with supraspinatus tendinopathy, both right after treatment and at one-year follow-up.

## 1. Introduction

Up to 64.3% of disorders of the shoulder area are caused by tendinopathies of the rotator cuff [1]. Among these, supraspinatus tendinopathy is the leading cause of shoulder pain in patients over 35 years old [2]. This condition is present both in male (4.5% of general population) and female (6.1% of general population) patients, and affects the stability of the shoulder joint complex. Additionally, important functional limitations are induced by pain [3]. 

Regarding evidence on treatment for supraspinatus tendinopathy, eccentric exercises are considered the gold standard followed by manual therapy [4], although their effects may be similar to those of glucocorticoid injection and arthroscopic subacromial decompression [5]. However, this finding is based on low-quality research [5]. Judging by the recent study of Cook et al. [6], corticosteroid injections may be beneficial in the short term (up to eight weeks) when compared with local anesthetic injections to manage rotator cuff-related shoulder pain (RCRSP). According to Diercks et al., there is no strong evidence on whether surgery may be more effective than conservative treatments [7]. Nevertheless, it would be recommended if other treatments failed [7,8].

Abdulla et al. suggests that supervised and home-based progressive shoulder strengthening and stretching are effective for the management of subacromial pain [9]. Eccentric exercise may reduce pain and improve strength in the management of shoulder pain and tendinopathies [10], while stabilization training of the scapula may also be recommended for subacromial pain syndrome [7].

Another technique currently recommended for the management shoulder pain in supraspinatus tendinopathy is Trigger point Dry Needling (TDN). This technique involves dry needling a taut band of a muscle in order to cause a “local twitch response”, which is an involuntary local muscle contraction induced by a spinal reflex [11]. The effects of this therapy include an increase in muscle blood flow and oxygenation, the suppression of spontaneous electrical activity in the area, the release of endogenous opioids, segmental inhibition induced by Gate Control mechanisms, and the release of inflammatory mediators [11]. This approach alone has shown good clinical results in shoulder and neck pain improvement in the short and medium terms [12,13,14] but also when delivered along with an exercise program [15]. Moreover, a recent study of Arias-Buría et al. supports its cost-effectiveness for the management of subacromial pain syndrome [16].

Percutaneous electrolysis (PE) is an electrotherapy technique that applies galvanic current through the skin using an echo-guided acupuncture needle [17]. This acts as a mediator to transfer electricity to the tissue to be treated [17]. During the emission phase, this needle will act as a negative electrode. Another electrode placed on the patient will act as the positive electrode [17,18]. Its mechanism of action is electrolysis, or the separation of ions whose charge is opposite to that of the tissue under treatment. This produces a “controlled” injury and an acute inflammatory response leading to the formation of new, healthy tissue [19].

There are few studies on the effectiveness of PE [3,17,18,20]. Furthermore, only two studies approach the treatment of pain in conditions affecting the shoulder [3,20]. According to Arias-Buría et al. [3], the treatment of shoulder pain with eccentric exercises and PE yields slightly better results than only eccentric exercises, but their benefits do not extend to function. In addition, short follow-up does not allow for the proper examination of the evolution of symptoms. In the study carried out by de-Miguel-Valtierra et al., a protocol of shoulder joint mobilization and eccentric exercises was compared with the same protocol combined with five sessions of PE at the supraspinatus tendon [20]. Follow-ups at three and six months were conducted, which showed no differences between groups concerning shoulder disability and pressure pain sensitivity. Therefore, the effects of PE on pain and function in patients with supraspinatus tendinopathy remains unclear.

PE seems to be a clinically effective tool when combined with eccentric exercise to rapidly and durably reduce symptoms [3,20], as it acts directly on the tissue composition of the damaged region [3,20]. However, more studies are needed about such a highly prevalent condition, particularly given that dry needling has been scientifically validated while the use of PE for this aim has little supporting evidence.

For these reasons, the aim of this trial was to compare the effects of PE with those of a well-established technique such as trigger point dry needling for the management of supraspinatus tendinopathy in patients treated with eccentric exercises. The hypothesis of our work was that treatment by PE is better than TDN in relieving pain and increasing the pressure pain threshold (PPT) and range of motion (ROM) of patients with supraspinatus tendinopathy who are concurrently subjected to eccentric exercise.

## 2. Materials and Methods

### 2.1. Study Design

The study was a single-blinded, prospective and longitudinal randomized clinical trial, conducted in accordance with the Helsinki Declaration and approved by the Ethics Committee of Servicio Andaluz de Salud. The trial is registered at ClinicalTrials.gov: identifier, NCT03184181; registry completion, April 2019. The reporting of data complies with the Consolidated Standards of Reporting Trials (CONSORT) statement.

### 2.2. Participants

In this study, from a selection of 40 adults, finally, 36 participants aged 25–60 fulfilled the following inclusion criteria: they were diagnosed with supraspinatus tendinopathy by obtaining positive results in the Hawkins–Kennedy test, a painful arc test during abduction [20,21] and ultrasound confirmation by the observation of tendon thickening with diffuse hypoechoic areas without tear [7] (performed by a medical doctor with over 20 years of experience in ultrasound diagnosis). Additionally, they presented active pain and hyperalgesia to algometer compression (2 kg/cm^2^) in the insertion zone of the tendon and the humerus when compared with the other side. This pain did not improve when using pharmacology or manual therapy. Patients who had previously undergone any surgical intervention at the arm were excluded from the study, as were those presenting any antecedents of fracture or luxation, those who had suffered from any severe disorder in the month prior to this study, those who had with bilateral affectation, and those who suffered from any cervical radiculopathy or fibromyalgia or who presented any contraindication related to the use of any of the techniques described in this study (i.e., pregnant women, cardiac pacemaker users, oncology patients, or those presenting any active infection or generalized lymphedema).

### 2.3. Randomization and Blinding

Participants were recruited from November of 2017 to January of 2018 in Clínica Santa María, Cádiz, Spain. One of the physiotherapists and the traumatologists held an informative session with all potential candidates in a physical medicine and rehabilitation center where the treatments took place. There, they received comprehensive information about the specifics of the study, as well as about the possible benefits and risks of the interventions. Once informed, participants signed an informed consent form that contained the previously mentioned information as well as their right to withdraw from the study at any moment and without having to justify their reasons.

Participants were randomized and assigned to either group, using the Epidat 4.1 software program, with an equal distribution (1:1). The assessing physician remained blind to this allocation (TDN or PE) thanks to the help of opaque envelopes prepared by a different researcher who was at the same time blinded for data collection and intervention. Follow-up was performed at one month and at one year.

### 2.4. Sample Size Calculation

Calculation of the sample size was based on the data provided for Arias-Buría et al. [3]. From these data, to obtain a difference of 1.1 points in the Numerical Pain Rating Scale (NPRS) with a power of 80% and a confidence level of 95%, a minimum of 16 patients per group are necessary. Finally, in order to improve the power, 18 patients were assigned to each group, to a total of 36. This calculation was performed using the software Ene version 3.0, by the Autonomous University of Barcelona.

### 2.5. Measurements

Pain: This was the main outcome of this study. This was measured using the Numeric Pain Rating Scale (NPRS), which assigns a number between 0 (lack of pain) and 10 (the worst pain) to quantify its intensity [22]. The NPRS showed an Intraclass Correlation Coeficient (ICC) of 0.61 to 0.95, a Standard Error of Measurement (SEM) of 0.48 to 1.02 and a Minimum Detectable Change (MDC) of 1.33 to 2.8 points in patients with musculoskeletal problems [23,24]. The patients were asked to use the NPRS to gauge the pain they were feeling at that exact moment.

Pressure pain threshold: The amount of pressure applied until the subject referred to pain for first time [25]. According to this, the sensitivity of the subacromial region and of distal, middle and proximal regions of the supraspinatus muscle were also considered, locating mechanosensible points on the upper trapezius close to the anatomical projection of supraspinatus trigger points [14] and pressing on them with an algometer until pain was felt. The value of the pressure reached was collected as the threshold where pain appeared. The reliability of this method was evaluated, showing a fair-to-excellent reliability [25]; the ICC ranged 0.78–0.93, and the coefficient of variation ranged from 10% to 22% [26].

Range of motion: Another variable measured was shoulder joint motion. For this purpose, a digital inclinometer was used (Baseline^®^, India), which is a tool validated for this purpose [27]. Shoulder flexion, extension, internal rotation and external rotation and the abduction ranges of motion where measured. These measures showed good reliability and an MDC of 4° to 9°, confirming that the change was not due to inter-trial variability or measurement error [27].

The variables were measured just before treatment and just after the last treatment session, and again as a follow-up at one month and at one year post-treatment.

### 2.6. Interventions

**Figure 1:**
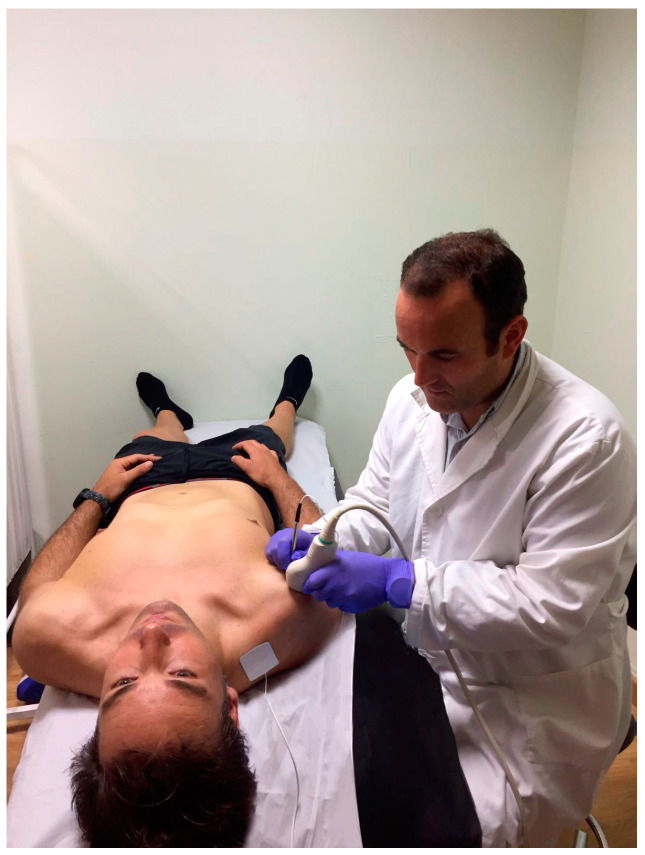
Application of percutaneous electrolysis (PE).

PE group: One treatment per week over four weeks (four sessions in total) using a percutaneous electrolysis EPTE^®^ device (Ionclinics & Deionic S.L., Valencia, Spain) at an intensity of 350 μA for 1.2 min [3,20] was performed. PE was applied on the injured zone of the supraspinatus tendon, which was located by ultrasound (Voluson 730 pro, General Electric^®^, Boston, MA, Massachusetts, USA) (Figure 1). For this purpose, the participant was placed in the supine position with the shoulder-to-treat in internal rotation. Isopropyl alcohol was used to sterilize the skin and a sonographic transducer enclosed in a sterile cover. Gel was applied between the sonographic transducer and the skin on the anatomical projection of the supraspinatus tendon. An acupuncture needle was inserted in the skin towards the supraspinatus tendon at an 80° angle. The same method has been used in other similar studies [3,20].

Trigger point dry needling group: A weekly session for four weeks (four sessions in total) of dry needling of the upper trapezius muscle towards supraspinatus muscle was held. A different researcher from the one of PE groups performed dry needling according to Hong’s fast-in and fast-out technique with the patient in the decubitus prone position. The upper member to be treated remained in a relaxed position along the body. A dermographic pencil was used to mark the location of the hyperalgesic points on the upper trapezius (on the anatomical projection of the supraspinatus muscle) that reproduced familiar pain to the patient and where pressure produced the characteristic local twitch response [14]. After that, the zone was disinfected with alcohol and the needle was inserted towards the supraspinatus fossa using hand gloves [14]. No other muscles were needled, to assure comparability with PE group, were only the supraspinatus was needled.

**Figure 2:**
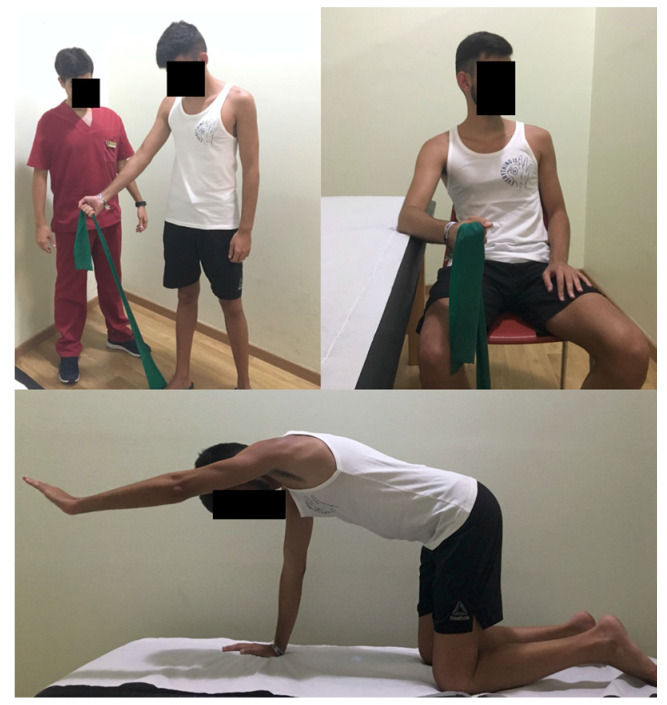
Performance of eccentric exercises for the supraspinatus.

Exercise program: As there is not a consensus about exercises for shoulder pain [28], patients of both groups were instructed in how to perform three different eccentric exercises used in other similar studies [3,15,20] for the supraspinatus that were to be performed daily at home from the first to the last day of treatment (3 × 10 repetitions):

Exercise for the supraspinatus: from standing position, the patient performed a shoulder abduction (concentric phase), followed by a slow shoulder adduction (eccentric phase). The patient used elastic bands to achieve proportional resistance. These were fixed to the floor using one foot (Figure 2).

Exercise for the infraspinatus: from the seating position and with the elbow resting on the treatment table, the patient performed an external rotation of the shoulder (concentric phase). The initial position was then slowly restored towards an inner rotation (eccentric phase). Resistance was provided by an elastic band, fixed to the floor with one foot (Figure 2).

Exercise for scapular global stabilization: in a quadruped position, the patient performed maximal shoulder flexion with an extended elbow (concentric phase) and returned to rest (eccentric phase) (Figure 2).

Patients were instructed for 15 min in the first intervention session and were then supervised and corrected when necessary in the following treatment sessions so as to ensure an adequate performance of the exercises mentioned above. They were told not to exceed three points on numeral pain rating scale (where 0 means “no pain”, and 10, “the worst pain possible”) while performing the exercises. Whenever pain exceeded three points on the scale during the exercise, a softer elastic band was used.

### 2.7. Statistical Analysis

Data were described using means and standard deviations for the continuous variables and by frequencies and percentages for the categorical ones. The Kolmogorov–Smirnov test was used to measure the normality of the distributions of the continuous variables. The chi-squared test and Student’s *t* test were used to analyze the baseline comparability of the groups. A 2X4 mixed-model analysis of variance (ANOVA) was used to examine the effects of the treatment conditions (PE versus TDN), as the between-subjects variable, and time (baseline, post-treatment, 1 month and 1 year) on the outcome variables. The NPRS at baseline was used as a covariable. The hypothesis of interest was the time-by-group interaction. Eta-squared was calculated to determine the effect size of the group-by-time interaction. Eta-squared is the equivalent in experimental studies of the coefficient of determination R^2^ and could be interpreted as the proportion of the between-groups differences dues to the effect of the differing treatments. According to Cohen, eta-squared can be deemed insignificant when <0.02, small if between 0.02 and 0.15, medium if between 0.15 and 0.35, and large if >0.35 [29]. Additionally, if a significant interaction was identified, pairwise Bonferroni comparisons were performed to explore the between-group differences at each time point. SPSS Statistical Package for Social Sciences, version 21.0 for Windows (SPSS Inc, Chicago, IL, USA) and MedCalc 18.2.1 (MedCalc, Belgium) were the two software programs used. Results were considered statistically significant at P-values below 0.05.

## 3. Results

**Figure 3:**
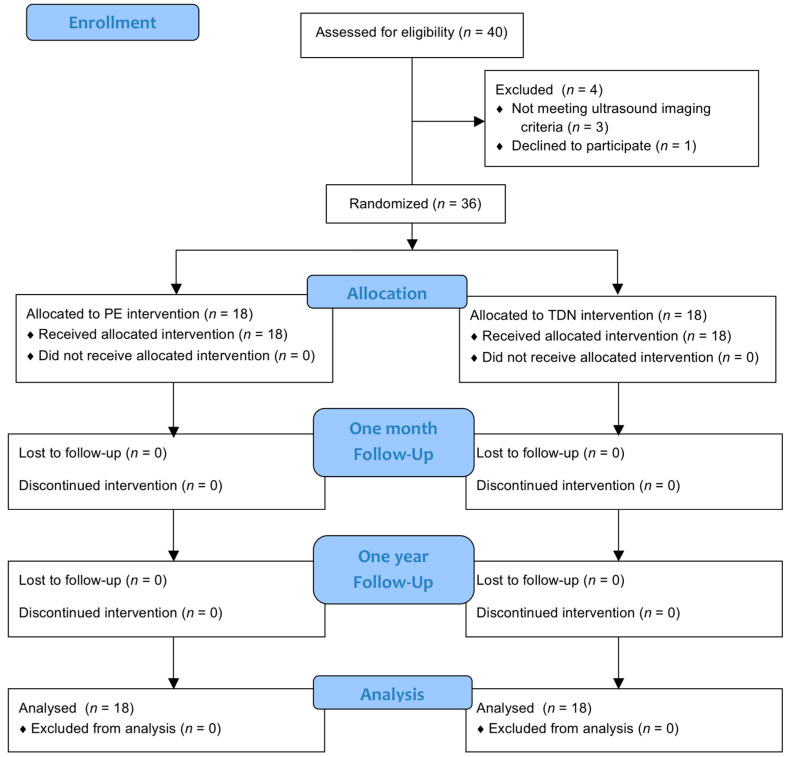
Flow diagram.

Of the 36 participants randomized, all completed the treatment and the follow-up (Figure 3).

The groups were comparable concerning demographic, morphologic and outcome variables at baseline except for NPRS (Table 1), which shows significant differences (*p* = 0.025), and for height, which shows differences in the limit of significance (*p* = 0.046).

**Figure 4:**
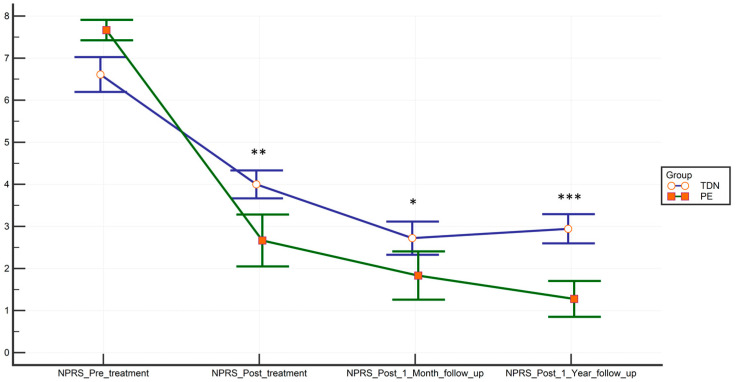
Time-point-dependent reduction of pain measured with the NPRS. Bars represent means and standard errors. * *p* < 0.05; ** *p* < 0.01; *** *p* < 0.001.

A linear mixed model revealed a significant time-by-group interaction that favored the PE group for flexion, extension, external rotations, NPRS, and all PPTs. There were no significant differences in abduction or internal rotation of the shoulder (Table 2). The effect size was medium for NPRS and shoulder flexion, and large for the proximal and middle supraspinatus mechanosensibility PPTs. The change in NPRS scores is shown in Figure 4.

Table 3 shows within-group differences at each time-point measurement for each outcome variable. The improvement was statistically significant at all time-point measurements and all the outcome variables of the PE (*p* < 0.001). As for the TDN Group, there were no significant changes at several time-point measurements concerning the mechanosensibility at hyperalgesic points of both middle and distal supraspinatus region PPTs.

Table 4 shows the Bonferroni’s pairwise comparison at each follow-up time point for the outcomes variables. They reveal significant between-groups differences that favor the Electrolysis Group, at all follow-up time-points, except for cervical flexion at the end of treatment.

## 4. Discussion

This work aimed to analyze the differences between PE and TDN for the improvement of pain, PPTs and ROM in patients with supraspinatus tendinopathy undergoing treatment with eccentric shoulder exercises. The results of our study show more significant improvements in the group treated with PE than in that treated with TDN, with a large effect size for the differences in the PPTs of the proximal and middle hyperalgesic regions of the supraspinatus, and medium, for the pain measured with the NPRS and for shoulder flexion and the distal trigger point PPT. Despite some variables, including pain, showing between-group significant differences, the magnitude of these differences was not very high, being close to the MDC. It also must be considered that the decision of only needling the supraspinatus to obtain a greater comparability among interventions could have reduced the effects of TDN from those obtained in clinical practice, with the additional needling of other trigger points in related muscles.

Although the results of the present study seem to be encouraging, studies on PE are still scarce, and we must exert caution before generalizing [3,17,18,20]. Moreover, the short follow-up period of previous studies as well as the lack of comparison with other techniques severely limits their scientific relevance [3,17,20]. For that reason, this study used a comparison group that underwent trigger point dry needling of the upper trapezius towards the supraspinatus muscle, which is an useful and cost-effectiveness treatment as proven by several authors [14,16]. The base treatment used was a protocol of eccentric exercises whose results have also been assessed [9]. Additionally, a long-term follow-up of one year was conducted to avoid some of the methodological limitations of other studies [3,20].

The results of this study support those observed by Arias et al. [3]. A similar treatment protocol was followed, but, in this case, a trigger point dry needling comparison group was established [12], and a follow-up was conducted one year after treatment to assess evolution. In these follow-ups, significant differences still favored the PE group concerning joint and pain sensitivity variables, both a month and a year after treatment. The results of the PE group agree with those found by Miguel Valtierra et al. [20], although they did not observe differences in pressure pain when comparing PE with manual therapy. This may be explained by the fact that the manual therapy protocol used by these authors was more effective than TDN or be because their patients received one more treatment session than the ones in this study (five versus four sessions) [20]. More studies will be necessary to assess if lower frequencies of treatment would produce similar results.

Regarding function, both TDN and PE produced significant improvements in the range of motion in patients with supraspinatus tendinopathy. A comparison between both groups showed significant higher improvements for PE in flexion, extension and external rotation. Patients in the PE group reached the necessary 130° in abduction and 142° in flexion to perform most of daily activities [30], while the TDN patients got very close. The findings agree with those about function in the study of Arias-Buría et al. [3].

The data yielded by this study are similar to those obtained when using ultrasound-guided PE to treat other disorders such as epicondylitis [17] and patellar tendinopathy [18]. Nevertheless, this study has allowed us to verify that improvement is significant in comparison with that achieved by other therapies, even when the control group received a treatment whose effectiveness had already been asserted. This was the limitation presented by prior research [3].

There are several studies that include information on the potential risk of possible vasovagal reactions [31,32,33]. Such reactions were not observed in any of the participants in this study. According to the same authors [31], these may be widely justified by the effect of needling and not only by that of the current. In spite of this, non-invasive treatments such as eccentric exercises should be the first option in the approach to supraspinatus tendinopathy, combining them with PE in case poor improvements are achieved.

### Limitations

Finally, this study presents several limitations. Firstly, a placebo group would be required to measure any improvement. However, there are remarkable methodological and ethical difficulties in applying invasive interventions as placebo, and for that reason, the comparison with a placebo group has not been considered viable. Additionally, a higher number of result variables would reveal more valuable information on the effects of this treatment, especially those related to shoulder function. Future studies should include other measures of function and pain as outcomes to check the effect of this treatment on these variables. Moreover, recording the exercise that participants performed along the follow-up period would have been useful to assess its relation to the improvements obtained and the adherence. It also must be considered that in clinical practice, not only the supraspinatus muscle is needled; other rotator cuff muscles are also treated, which could present trigger points. Regarding medication intake, participants were asked to avoid any change in their common treatments in order to avoid possible side effects and/or alterations in their usual pain. Finally, it must be kept in mind that both groups performed an eccentric exercise protocol and that further studies should look into how much of the improvement is due to that intervention.

## 5. Conclusions

Eco-guided percutaneous electrolysis may be more effective, both in the short- and long-term, than trigger point dry needling in improving the shoulder pain, range of motion and PPTs of a group of supraspinatus tendinopathy patients treated with eccentric exercise.

## Figures and Tables

**Table 1:** Clinical characteristics and baseline comparability of both groups.

		All	*N* = 36	TDN	*n* = 18	PE	*n* = 18	*p*-Value
		Count	%	Count	%	Count	%	
Gender *	Male	27	75.0	11	61.1	16	88.9	0.054
	Female	9	25.0	7	38.9	2	11.1	
Affected Side *	Right	26	72.2	14	77.8	12	66.7	0.457
	Left	10	27.8	4	22.2	6	33.3	
Dominant Side *	Right	35	97.2	18	100.0	17	94.4	0.31
	Left	1	2.8	0	0.0	1	5.6	
		**Mean**	**SD**	**Mean**	**SD**	**Mean**	**SD**	
Age		40.04	9.88	40.92	8.40	39.17	11.36	0.601
Height		1.74	0.05	1.72	0.05	1.75	0.05	0.046 *
Weight		80.09	11.26	78.45	13.70	81.72	8.22	0.391
BMI		26.72	3.79	26.79	4.67	26.64	2.78	0.906
NPRS		7.11	1.52	6.55	1.74	7.67	1.03	0.025 *
Flexion		123.89	16.97	129.17	18.31	118.61	14.10	0.061
Extension		25.74	6.72	24.36	5.58	27.11	7.61	0.224
Internal rot.		63.15	16.03	59.58	12.30	66.72	18.72	0.185
External rot.		63.11	17.82	60.83	16.16	65.39	19.53	0.450
Abduction		102.69	17.06	98.61	15.77	106.78	17.76	0.154
Prox PPT		2.30	0.72	2.27	0.76	2.33	0.71	0.803
Med PPT		2.57	0.89	2.59	0.73	2.54	1.05	0.871
Distal PPT		2.49	0.89	2.55	0.74	2.43	1.04	0.682

TDN: Trigger point Dry needling; PE: Percutaneous Electrolysis; Ext: external; Int: internal; NPRS: Numerical Pain Rating Scale; PPT: Pressure Pain Threshold; P: proximal; M: middle; D: distal; * Categorial variables are described by frequencies and percentages. The rest of the variables are described by mean and standard deviation (SD). *p*-values correspond to the chi-squared test for categorical variables and t-test for continuous variables. * *p* < 0.05.

**Table 2:** Data description, statistical significance and effect size of the time-by-group interaction from the analysis of variance with NPRS at baseline as a covariable.

	TDN Group		PE Group				
	Mean	SD	Mean	SD	*p*-Value	ETA2	Effect
Abd_pre	98.61	15.77	106.78	17.76	0.058	0.079	Small
Abd_post	124.84	18.39	138.22	17.73			
Abd_1M	129.72	18.97	141.00	17.17			
Abd_1Y	124.17	18.48	140.72	11.93			
Flex_pre	129.17	18.31	118.61	14.10	<0.001 ***	0.334	Medium
Flex_post	143.99	20.58	152.22	12.56			
Flex_1M	146.01	20.24	155.50	10.27			
Flex_1Y	140.82	19.41	154.50	8.62			
Ext_pre	24.36	5.58	27.11	7.61	0.014 *	0.129	Small
Ext_post	30.50	4.09	37.39	5.28			
Ext_1M	33.09	3.79	37.56	4.63			
Ext_1Y	30.63	3.80	39.50	4.18			
Int_rot_pre	59.58	12.30	66.72	18.72	0.202	0.049	Small
Int_rot_post	69.91	10.95	86.89	9.66			
Int_rot_1M	73.29	10.54	87.44	9.51			
Int_rot_1Y	72.36	11.97	87.50	8.27			
Ext_rot_pre	60.83	16.16	65.39	19.53	0.048 *	0.102	Small
Ext_rot_post	70.33	12.27	86.83	5.88			
Ext_rot_1M	74.61	12.51	88.39	3.24			
Ext_rot_1Y	71.81	12.87	87.78	3.52			
NPRS_pre	6.55	1.74	7.67	1.03	0.002 **	0.188	Medium
NPRS_post	4.05	1.42	2.67	2.61			
NPRS_1M	2.77	1.66	1.81	2.44			
NPRS_1Y	2.93	1.46	1.28	1.81			
PPT_P_pre	2.27	0.76	2.33	0.71	<0.001 ***	0.353	Large
PPT_P_post	2.82	0.73	3.99	1.02			
PPT_P_1M	3.08	0.80	4.45	0.83			
PPT_P_1Y	3.11	0.62	4.75	0.60			
PPT_M_pre	2.59	0.73	2.54	1.05	<0.001 ***	0.378	Large
PPT_M_post	2.91	0.76	4.04	0.98			
PPT_M_1M	3.04	0.71	4.48	0.79			
PPT_M_1Y	3.09	0.75	4.73	0.55			
PPT_D_pre	2.55	0.74	2.43	1.04	<0.001 ***	0.281	Medium
PPT_D_post	3.03	0.75	3.90	1.09			
PPT_D_1M	3.16	0.64	4.42	0.89			
PPT_D_1Y	3.16	0.59	4.69	0.67			

TDN: Trigger point Dry needling; PE: Percutaneous Electrolysis; Ext: external; Int: internal; Rot: rotation; NPRS: Numerical Pain Rating Scale; PPT: Pressure Pain Threshold; P: proximal; M: middle; D: distal; 1M: one month follow-up; 1Y: one year follow-up. * *p* < 0.05; ** *p* < 0.01; *** *p* < 0.001.

**Table 3:** Within-group differences compared to baseline.

Variable	Time	TDN					PE				
		Diff.	SE	95% CI			Diff.	SE	95% CI		
				Lower	Upper	*p*			Lower	Upper	*p*
ABD	Post	25.43	3.47	15.69	35.16	<0.001	32.24	3.47	22.51	41.98	<0.001
	Post-1M	30.26	3.53	20.37	40.16	<0.001	35.07	3.53	25.18	44.97	<0.001
	Post-1Y	23.47	3.82	12.75	34.18	<0.001	36.04	3.82	25.33	46.75	<0.001
Flexion	Post	15.44	3.51	5.60	25.29	0.001	32.99	3.51	23.14	42.83	<0.001
	Post-1M	17.28	2.87	9.21	25.34	<0.001	36.45	2.87	28.38	44.52	<0.001
	Post-1Y	11.94	3.13	3.16	20.73	0.003	35.60	3.13	26.82	44.39	<0.001
Extension	Post	6.50	1.05	3.55	9.45	<0.001	9.92	1.05	6.97	12.87	<0.001
	Post-1M	9.32	1.25	5.83	12.82	<0.001	9.85	1.25	6.36	13.35	<0.001
	Post-1Y	6.77	1.56	2.39	11.14	0.001	11.89	1.56	7.52	16.27	<0.001
Internal	Post	11.48	3.37	2.00	20.95	0.011	19.02	3.37	9.54	28.49	<0.001
Rotation	Post-1M	14.63	3.54	4.71	24.56	0.001	19.79	3.54	9.86	29.72	<0.001
	Post-1Y	13.27	3.56	3.27	23.27	0.004	20.28	3.56	10.29	30.28	<0.001
External	Post	9.94	3.28	0.74	19.14	0.028	21.01	3.28	11.80	30.21	<0.001
Rotation	Post-1M	14.46	3.56	4.46	24.45	0.002	22.33	3.56	12.33	32.32	<0.001
	Post-1Y	11.42	3.48	1.64	21.20	0.015	21.96	3.48	12.18	31.73	<0.001
NPRS	Post	−2.75	0.49	−4.11	−1.39	<0.001	−4.75	0.49	−6.11	−3.38	<0.001
	Post-1M	−4.02	0.48	−5.36	−2.68	<0.001	−5.63	0.48	−6.96	−4.29	<0.001
	Post-1Y	−3.88	0.36	−4.90	−2.86	<0.001	−6.12	0.36	−7.15	−5.10	<0.001
PPT_P	Post	0.54	0.18	0.02	1.06	0.039	1.67	0.18	1.15	2.19	<0.001
	Post-1M	0.79	0.20	0.23	1.36	0.002	2.13	0.20	1.56	2.69	<0.001
	Post-1Y	0.84	0.16	0.39	1.29	<0.001	2.41	0.16	1.96	2.86	<0.001
PPT_M	Post	0.30	0.20	−0.25	0.86	0.784	1.51	0.20	0.95	2.06	<0.001
	Post-1M	0.46	0.21	−0.12	1.03	0.197	1.92	0.21	1.35	2.50	<0.001
	Post-1Y	0.50	0.20	−0.06	1.06	0.108	2.19	0.20	1.62	2.75	<0.001
PPT_D	Post	0.49	0.24	−0.18	1.16	0.288	1.46	0.24	0.79	2.13	<0.001
	Post-1M	0.62	0.23	−0.03	1.28	0.072	1.97	0.23	1.31	2.63	<0.001
	Post-1Y	0.65	0.22	0.04	1.27	0.032	2.23	0.22	1.61	2.84	<0.001

TDN: Trigger point Dry needling; PE: Percutaneous Electrolysis; NPRS: Numerical Pain Rating Scale; PPT: Pressure Pain Threshold; P: proximal; M: middle; D: distal; Diff: Mean differences; SE: Standard Error; CI: Confidence Interval; 1M: one month later; 1Y: one year later; *p*: *p*-value.

**Table 4:** Between-group pairwise comparison of Bonferroni at each follow-up point for significant variables from the Linear Mixed Model.

	Post-Treat		95% CI			1 Month		95% CI			1 Year		95% CI		
	Diff	SE	Low	upp	*p*	Diff	SE	Low	upp	*p*	Diff	SE	Low	upp	*p*
Flex	10.49	6.13	−1.98	22.95	0.096	12.12	5.72	0.47	23.76	0.042	16.60	5.30	5.81	27.39	0.004
Ext	7.25	1.71	3.77	10.74	<0.001	4.36	1.54	1.22	7.49	0.008	8.96	1.45	6.00	11.92	<0.001
Ext rot	19.27	3.26	12.64	25.90	<0.001	16.08	3.15	9.66	22.49	<0.001	18.74	3.19	12.26	25.23	<0.001
NPRS	−1.99	0.71	−3.44	−0.55	0.008	−1.61	0.70	−3.03	−0.18	0.028	−2.24	0.53	−3.33	−1.16	<0.001
PPT_P	1.31	0.32	0.67	1.96	<0.001	1.52	0.29	0.93	2.11	<0.001	1.76	0.22	1.32	2.20	<0.001
PPT_M	1.23	0.32	0.58	1.87	<0.001	1.49	0.27	0.93	2.05	<0.001	1.71	0.24	1.23	2.19	<0.001
PPT_D	1.00	0.34	0.32	1.68	0.005	1.38	0.28	0.81	1.94	<0.001	1.60	0.23	1.14	2.06	<0.001

NPRS: Numerical Pain Rating Scale; PPT: Pressure Pain Threshold; P: proximal; M: middle; D: distal; Diff: Mean differences; SE: Standard Error; CI: Confidence Interval; Treat: Treatment; Low: lower difference; upp: upper difference; *p*: *p*-value.
